# Identification of Reference Genes for Circadian Studies on Brain Microvessels and Choroid Plexus Samples Isolated from Rats

**DOI:** 10.3390/biom11081227

**Published:** 2021-08-17

**Authors:** Aleksandra Szczepkowska, András Harazin, Lilla Barna, Mária A. Deli, Janina Skipor

**Affiliations:** 1 Institute of Animal Reproduction and Food Research, Polish Academy of Sciences, 10-748 Olsztyn, Poland; j.skipor@pan.olsztyn.pl; 2 Institute of Biophysics, Biological Research Centre, 6726 Szeged, Hungary; harazin.andras@brc.hu (A.H.); barna.lilla@brc.hu (L.B.)

**Keywords:** reference genes, circadian rhythm, brain barriers, brain microvessels, choroid plexus, qPCR

## Abstract

Delivery of putative compounds of therapeutic value to the brain is limited by brain barriers: the blood–brain barrier located in the endothelium of the brain microvessels (BrMV) and the blood–cerebrospinal fluid barrier located in the epithelium of the choroid plexus (ChP). Understanding their function and modulation by the circadian clock may enhance the efficacy of brain-targeting therapies. The aim of the present study was to evaluate the stability of 10 reference genes in the BrMV and ChP, isolated from male and female rats at six time points (ZT1, 5, 9, 13, 17, and 21). Gene evaluations were performed by qPCR, analyzed by RefFinder tool, and verified by analyzing the expression of the brain and muscle ARNT-like 1 (*Bmal1*) using the qPCR and digital PCR methods. We identified as the most stable genes for circadian studies tyrosine 3-monooxygenase/tryptophan 5-monooxygenase activation protein zeta (*Ywhaz*) and apolipoprotein E (*Apoe*) for BrMV, and beta actin (*Actb*) and hypoxanthine-guanine phosphoribosyltransferase (*Hprt1*) for ChP. After verification, ribosomal protein (*Rps18*) was also included as a sufficient reference gene. Additionally, the observed gender difference in the *Bmal1* oscillations in both BrMV and ChP suggests that separate studies for each gender are recommended.

## 1. Introduction

The highly selective blood–brain barrier (BBB) is located in the brain microvascular endothelium (BrMV), which, along with pericytes, perivascular astrocytes, microglia, and neurons, create the neurovascular unit (NVU) [1]. The blood–cerebrospinal fluid barrier (BCSFB) is located in the choroid plexus (ChP) epithelium. The BBB and BCSFB tightly regulate the movement of ions, molecules, and cells between the blood and the brain [2]. These barriers, from one side, allow proper neuronal function and protection of the neural tissue from toxins and pathogens, but, from the other side, restrict the entry of many drugs from the blood into the brain [1,3]. Therefore, a significant amount of research capacity is devoted to developing new strategies for the delivery of therapeutic compounds to the brain [4,5].

In recent years, a growing body of evidence demonstrated that circadian rhythms and sleep/awake cycles may affect the function of the brain barriers [6,7]. The physiological regulation of circadian rhythms involves a master circadian pacemaker in the suprachiasmatic nucleus and numerous peripheral oscillators. Almost all tissues have their own circadian clock, peripheral clocks that oscillate independently even after organs are removed from the organism [8]. The molecular mechanisms underlying circadian regulation are cell-autonomous transcription-translation feedback loops. In simple terms, the complex of transcription factors BMAL1 (brain and muscle ARNTlike 1) and CLOCK (circadian locomotor output cycles kaput) or NPAS2 (neuronal PAS domain-containing protein 2) activates the expression of Period (Per1/2) and Cryptochrome (Cry1/2), which represent negative elements of the loop, and their protein products inhibit the activity of BMAL1/CLOCK or NPAS2 complexes, hence their own expression. The additional loop is created by REVERβ (nuclear receptor subfamily 1 group D member 1) and RORs (retinoid-related orphan receptors), transcription factors that negatively (REVERβ) and positively (RORs) regulate the expression of BMAL1. Finally, these factors, acting in positive and negative loops, regulate the expression of thousands of clock-controlled genes that drive tissue-specific circadian output in physiology; for review, see [9]. In the rat, ChP rhythmic expression of BMAL1, CRY2, PER1, and PER2 was observed [10], and ChP has been demonstrated to function as an important circadian clock component [7], and a recent review showed clearly the impact of circadian rhythm on the function of ChP [11]. Moreover, in the rat brain endothelial cells, Carver et al. [12] showed daily fluctuations in the expression of BMAL1 and REVERBα. Studies by Nakazato et al. [13] showed that, in mice, deletion of *Bmal1* gene causes a significant increase in BBB permeability. Moreover, in 2018, Zhang et al. [14] published the groundbreaking discovery that the Drosophila brain barrier has a molecular clock that renders it more or less permeable during certain hours of the day. All these data suggest the necessity to consider chronobiology in the new strategies of drug administration to the brain. However, for that purpose, many basic analyses considering the circadian transcriptional changes among brain barriers are necessary.

For analysis of the transcriptomic changes in ChP and BrMV, considered as models of the brain barriers, real-time quantitative RT-PCR (qPCR) seems to be the first methodological choice. qPCR is the dominant quantitative technique thanks to its broad dynamic range, accuracy, sensitivity, specificity, and speed [15]. However, relative quantification of qPCR data requires the use of reference genes for normalization. It should be kept in mind that the normalization of qPCR analysis can often affect the final result and the conclusions drawn from it. Unfortunately, there is no universal reference gene that is stably expressed under all experimental conditions and in all tissue types. Specifically, circadian studies require the selection of reference genes whose expression does not oscillate around the clock. For that purpose, we examined 10 reference genes using the RefFinder tool, which combines four different algorithms: ΔCt, BestKeeper, Genorm, and NormFinder. We compared reference genes’ stability in ChP and BrMV, isolated over the clock at six time points, and verified genes selected as the most stable by analyzing the expression of *Bmal1* (brain and muscle ARNT-like 1) gene using qPCR and digital PCR methods.

## 2. Materials and Methods

### 2.1. Animals and Tissue Collection

**Figure 1:**
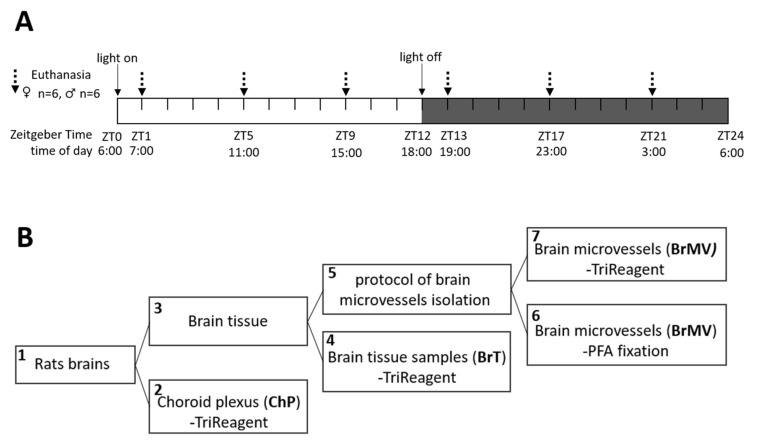
Schematic diagram of the experimental design: (**A**) and collection of the brain cortex tissue (BrT), choroid plexus (ChP), and brain microvessels (BrMV, **B**). The dashed arrows in the upper panel (**A**) indicate the time points at which the animals were euthanized.

All procedures were performed in compliance with the relevant laws and institutional guidelines for the care and use of animals in research according to the Directive 2010/63/EU. Studies were conducted on 12-week-old male (*n* = 36, 400–500 g) and female (*n* = 36; ~300 g) Wistar rats maintained in artificial light conditions with a 12 h/12 h light/dark cycles, with standard nutrition and water ad libitum. Only females in the diestrus phase of the estrous cycle (verified by microscopic observation of vaginal swabs according to Marcondes et al. [16]) were selected. Animals were euthanized by an overdose of pentobarbital (160 mg/kg body weight, intraperitoneal injection) at six time points (*n* = 12/each time point; *n* = 6 females and *n* = 6 males, according to the schedule presented in Figure 1A) and then immediately decapitated. During the dark phase, all manipulations were carried out under red light. Heads were placed into sterile Petri dishes kept on ice. The isolation of brain, ChP, and BrMV samples was performed in sterile conditions according to the schematic diagram presented in Figure 1B.

In **step 1** of the procedure (Figure 1B), immediately after decapitation, the olfactory bulb and cerebellum were removed and brain cortical samples were collected and placed into a sterile Petri dish, kept on ice and filled with sterile PBS. In **step 2**, ChP from lateral ventricles was collected according to the method presented by Bowyer et al. [17], and ChPs collected from two animals were pooled as one sample and then directly dissolved in TRI Reagent (Sigma-Aldrich, Merck, Darmstadt, Germany), frozen, and stored at −80 °C. **Step 3**: Meninges and large vessels from the surface of brain cortical samples were removed and the tissue was transferred into a sterile Petri dish containing 1 mL Ringer-HEPES (RH) and kept on ice. **Step 4**: Samples of brain tissue (BrT) were collected and pooled from two animals as one sample, directly dissolved in TRI Reagent, frozen, and stored at −80 °C. **Step 5**: The remaining brain tissue was pooled from two animals as one sample and then BrMV were isolated according to the protocol described by Yousif et al. [18]. Briefly, brain tissues were cut into very small pieces in the 1 mL RH buffer, then homogenized on ice in a final volume of 4 mL of ice-cold RH per one rat brain (8 mL per sample) with a Potter–Elvehjem PTFE homogenizer (Sigma-Aldrich, Merck, Darmstadt, Germany), using 20 up-and-down movements. The obtained homogenate was centrifuged at 1000× *g* for 10 min at 4 °C. The pellet was suspended in 10 mL (per sample) of 17.5% dextran (MW 64-76 kDa, Sigma-Aldrich, Merck, Darmstadt, Germany) in RH and centrifuged at 1500× *g* for 15 min at 4 °C. The resulting pellet was suspended in RH containing 1% bovine serum albumin (BSA, Sigma-Aldrich, Merck, Darmstadt, Germany), while the supernatant containing a layer of myelin was centrifuged once more. The two resulting pellets suspended in RH with 1% BSA were pooled as one sample. The obtained suspension was passed through a 100 µM nylon mesh to keep macrovessels, followed by passing through a 20 µM nylon mesh (PluriStrainer, PluriSelect Life Science, Leipzig, Germany). The BrMV retained by the 20 µM mesh was collected immediately and checked under a microscope; then a 10 µL volume was collected for validation; and the remaining part was divided into two parts, frozen, and stored at −80 °C. **Step 6**: The volume of 10 µL collected during each BrMV isolation was pooled and then fixed in 3% paraformaldehyde in PBS for 30 min, followed by two washes in PBS and suspension in 1% FBS-PBS. Thereafter, samples were kept at 4 °C until immunofluorescent analysis. **Step 7**: The remaining BrMV were dissolved in TRI Reagent, frozen, and stored at −80 °C.

### 2.2. RNA Extraction, cDNA Preparation, and qPCR Analysis

Frozen samples of ChP, BrMV, and BrT were thawed, and RNA was isolated according to the modified Chomczyński method [19]. During RNA isolation, the genomic DNA digestion step was carried out, using DNase I solution (Sigma-Aldrich, Merck, Darmstadt, Germany). The concentration and purity of the obtained RNA were evaluated spectrophotometrically using a NanoDrop 1000 instrument (Thermo Fisher Scientific, Waltham, MA, USA). RNA integrity was controlled by the electrophoresis method using 1.2% agarose gel containing the GelRed Nucleic Acid Gel Stain (Biotium, Fremont, CA, USA). Reverse transcription (RT) of total RNA was performed using DyNAmo cDNA Synthesis Kit (Thermo Fisher Scientific, Waltham, MA, USA), according to the manufacturer’s protocol, and each RT reaction contained 1 µg of total RNA. Obtained cDNA was frozen and kept at −80 °C until further analysis.

qPCR analysis was performed using the Viia7 instrument, using 384-well formats (Applied Biosystems, Thermo Fisher Scientific, Waltham, MA, USA). Each real-time PCR reaction contained 3 µL cDNA (1:10), 0.2 µM of each oligonucleotide from specific pair, and 5 µL of DyNAmo SYBR Green qPCR kit with ROX (Thermo Fisher Scientific, Waltham, MA, USA). The cDNA samples and reaction mix were transferred into 384-well plates, by the Bravo Automated Liquid Handling Platform (Agilent Technologies, Santa Clara, CA, USA). The following protocol was used: 95 °C for 10 min for the hot start modified Tbr DNA polymerase, and 40 cycles at 95 °C for 15 s (denaturation), 60 °C or 55 °C for 30 s (primer annealing), and 72 °C for 30 s (extension). After the cycles, a final melting curve analysis was performed under continuous fluorescence measurement to assess the specificity of the amplification. Expression analysis was performed using the Real-Time PCR Miner software (available online: http://ewindup.info/miner/) based on the algorithm of Zhao and Fernald [20] that determines the actual reaction efficiency for each pair of primers for every single reaction.

### 2.3. Verification of the Tissue Isolation Process

The verification of the ChP collection and BrMV proper isolation was based on qPCR according to the paper of Dai et al. [21]. For that purpose, the primers designed for plasmalemma vesicle-associated protein (*Plvap,* fenestration marker), claudin-5 (*Cldn-5*, BBB tight junction (TJ) marker), and endothelial cell adhesion molecule 1 (*Pecam1,* endothelial cell marker) were used in qPCR analysis carried out on cDNA derived from ChP, BrMV, and BrT collected at the first time point (ZT1). The specific primer pairs listed in Table 1 were selected based on literature data or designed by Primer-BLAST (National Center for Biotechnology Information, Bethesda, MD, USA) and synthesized by Genomed (Warsaw, Poland). For calculation of the qPCR results, the geometric mean of two reference genes: *Hprt1* and *Actb* (primers’ sequences in Table 1), was used. Additionally, the cellular composition of BrMV fractions was characterized by immunofluorescent analysis using primary antibodies against the cellular and molecular components of the neurovascular unit: CLDN-5 (rabbit polyclonal primary antibody, Cat. No. SAB4502981, Sigma-Aldrich, Merck, Darmstadt, Germany), PECAM1 (rabbit polyclonal primary antibody, Cat. No. SC1506R, Santa Cruz Biotechnology, Dallas, TX, USA), glial fibrillary acidic protein (GFAP, astrocytes marker; mouse monoclonal primary antibody, Cat. No. G3893, Sigma-Aldrich, Merck, Darmstadt, Germany), and platelet-derived growth factor receptor beta (PDGFRβ, pericytes marker; rabbit monoclonal primary antibody, Cat. No. ab32570, Abcam, Cambridge, UK). The pooled BrMV fractions were incubated overnight with primary antibody solutions at 4 °C, then incubated with appropriate secondary antibodies (anti-rabbit secondary antibody conjugated with Cy3, Cat. No. C2306, Sigma-Aldrich, Merck, Darmstadt, Germany; and anti-mouse secondary antibody labeled with Alexa Fluor 488, Invitrogen, Thermo Fisher Scientific, Waltham, MA, USA) and H33342 nucleus stain (Cat. No. 382065, Calbiochem, Merck, Darmstadt, Germany) for nuclei visualization. Leica TCS SP5 confocal laser scanning microscope (Leica Microsystems, Wetzlar, Germany) was used for visualization and archiving.

### 2.4. Identification and qPCR Verification of Optimal Reference Genes

Ten potentially suitable reference genes commonly used for qPCR data normalization were chosen as candidates (Table 1). The online tool RefFinder (available online at https://www.heartcure.com.au/reffinder (accessed on 15 August 2021), shared by Heart Cure Australia Biotechnology and Bioinformatics) was used to evaluate the expression stability of reference gene candidates by applying the four algorithms: Genorm [22], NormFinder [23], BestKeeper [24], and the ΔCt method [25]. The lowest stability value indicates the most stable expression. For qPCR analysis, the specific primers pairs for reference genes were selected based on the literature data. All primers were synthesized by Genomed (Warsaw, Poland) and their sequences and source data are listed in Table 1. Verification of those selected as the most and as the least stable reference genes was based on *Bmal1* relative expression, analyzed using the qPCR method.

**Table 1:** Sequences of oligonucleotide primers used for qPCR analysis.

	Gene	(Forward/Reverse) Sequence 5′→3′		Amplicon Size (bp)	References/Sources
***Reference genes***	***Actb***	F: TGTCACCAACTGGGACGATA	R: GGGGTGTTGAAGGTCTCAAA	165	[26]
	***Apoe***	F: CCTGAACCGCTTCTGGGATT	R: GCTCTTCCTGGACCTGGTCA	65	[27]
	***Hmbs***	F: TCCTGGCTTTACCATTGGAG	R: TGAATTCCAGGTGAGGGAAC	176	[26]
	***Hprt1***	F: CCTGTTGATGTGGCCAGTAAAGA	R: ATCAAAAGGGACGCAGCAAC	137	[28]
	***Pgk1***	F: ATGCAAAGACTGGCCAAGCTAC	R: AGCCACAGCCTCAGCATATTTC	104	[29]
	***Ppia***	F: TATCTGCACTGCCAAGACTGAGTG	R: CTTCTTGCTGGTCTTGCCATTCC	126	[29]
	***Rplp2***	F: ATTGAGGATGTCATCGCTCAGG	R: TCTTTCTTCTCCTCTGCTGCAG	137	[28]
	***Rps18***	F: ACGGACCAGAGCGAAAGCAT	R: TGTCAATCCTGTCCGTGTCC	310	[26]
	***Tbp***	F: TAATCCCAAGCGGTTTGCTG	R: TTCTTCACTCTTGGCTCCTGTG	111	[30]
	***Ywhaz***	F: TTCGCAGCCAGAAAGCAAAG	R: TTGTCATCACCAGCAGCAAC	87	[28]
***Marker genes***	***Plvap***	F: ATAATCGGTTCATCGCCGCT	R: GCTTGAAGAGCAAGGCTTCG	96	NM_020086.1
	***Cldn-5***	F: CTACAGGCTCTTGTGAGGACTTGAC	R: AGTAGGAACTGTTAGCGGCAGTTTG	121	[31]
	***Pecam1***	F: GCCTCACCAAGAGAACGGAA	R: AATTGGATGGCTTGGCCTGA	191	NM_031591.1
	***Bmal1***	F: TGGACTGCAACCGCAAGAG	R: CCTTCCATGAGGGTCATCTTTG	137	[28]

*Actb*—beta actin; *Apoe*—apolipoprotein E; *Hmbs*—hydroxymethylbilane synthase; *Hprt1*—hypoxanthine-guanine phosphoribosyltransferase; *Pgk1*—phosphoglycerate kinase 1; *Ppia*—peptidylprolyl isomerase A; *Rplp2*—ribosomal protein lateral stalk subunit P2; *Rps18*—ribosomal protein S18, *Tbp*—TATA-box binding protein; *Ywhaz*—tyrosine 3-monooxygenase /tryptophan 5-monooxygenase activation protein zeta; *Plvap—*plasmalemma vesicle associated protein (originally designed/ R:487/488 exon span)*; Cldn-5*—claudin 5; *Pecam1*—platelet and endothelial cell adhesion molecule 1 (originally designed/ F:2212/2213 exon span); *Bmal1*—brain and muscle ARNT-like 1 (clock gene).

### 2.5. Digital PCR

The actual copy number of Bmal1 mRNA was determined using digital PCR, carried out according to QuantStudio 3D Digital PCR System (Thermo Fisher Scientific, Waltham, MA, USA), based on sealed chip technology. This method was used to determined Bmal1 expression, for verification of reference genes selected for qPCR analysis. Each digital PCR reaction contained 5 µL BrMV/ChP cDNA (1:10) samples, 0.2 µM of each primer: F: 5’-TGGACTGCAACCGCAAGAG-3’ and R: 5’- CCTTCCATGAGGGTCATCTTTG-3’ as well as 900 nM of TaqMan probe: FAM- 5’- CCATACTTTCTTGGTAGTTCAGTGGACTGC-3’ -TAMRA [32], and the QuantStudio 3D Digital PCR V2 MasterMix (Thermo Fisher Scientific, Waltham, MA, USA). The primers and probe used were synthesized by Genomed (Warsaw, Poland). The results were analyzed in the QuantStudio 3D AnalysisSuite Cloud Software (Thermo Fisher Scientific, Waltham, MA, USA) and normalized. 

### 2.6. Statistical Analysis

Statistical analyses were performed with the Graph Pad PRISM 8 software (Graph Pad Software, San Diego, CA, USA). To analyze the circadian oscillation of *Bmal1* gene expression, CircWave v1.4 software (www.huttlab.nl, www.euclock.org an extension of the Cosinor analysis, courtesy of Dr. Roelof Hut) was used by fitting a cosine algorithm (R-squared, r^2^). The statistical significance, calculated by F-test, was considered when *p*-value < 0.05. For qPCR analysis, one-way ANOVA with Tuckey post-hoc test was performed to determine the differences in the relative expression of *Plvap*, *Cldn-5,* and *Pecam1* between three types of tissue: ChP, BrMV, and BrT, and to determine the differences between *Bmal1* mRNA expression analyzed by digital PCR and qPCR with using a different combination of selected reference genes. The results of qPCR are presented as the mean of relative gene expression (±SEM) versus the geometric mean of selected reference genes’ combination. The qPCR and digital PCR results were normalized by dividing all partial results by the median value from the ZT1 group (07:00); therefore, the values in the ZT1 group were brought close to 1. Statistical significance was assumed at *p* < 0.05.

## 3. Results

### 3.1. Characterization of the Isolated BrMV Fractions and ChP

**Figure 2:**
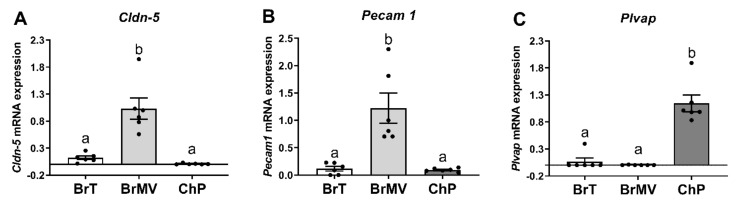
Mean (±SEM) relative mRNA expression of claudin-5 (*Cldn-5,* **A**), endothelial cell adhesion molecule (*Pecam1*, **B**), and plasmalemma vesicle-associated protein (*Plvap*, **C**) in brain tissue (BrT, white bars), brain microvessels (BrMV, grey bars), and choroid plexus (ChP, dark bars) isolated from rats brain (*n* = 6, 6 females/3 samples and 6 males/3 samples for each time point). Different lowercase letters indicate significant differences at *p* < 0.05.

The highest mRNA level of *Cldn-5* was observed in isolated BrMV, while its expression was not detectable in ChP and was very low in BrT (Figure 2A). The highest *Pecam1* mRNA level was observed in the BrMV fraction, whereas a very low expression level was detected in ChP and BrT (Figure 2B). In turn, *Plvap* had the highest mRNA level in ChP, while its expression was not observed in BrMV and was very low in BrT (Figure 2C). These data confirm the adequacy of the isolation and collection method to obtain BrMV fractions and ChP samples.

**Figure 3:**
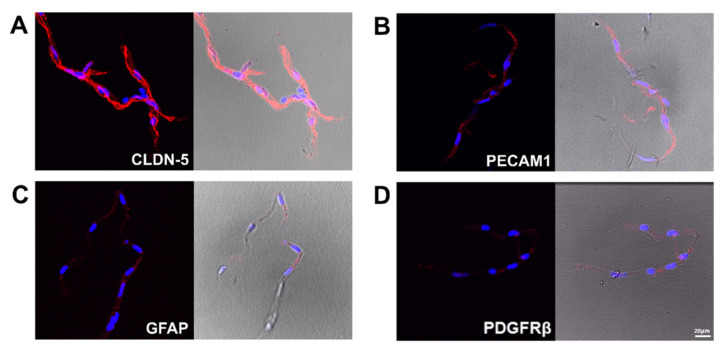
Immunostaining of brain microvessels fractions for markers of neurovascular unit cells presented as red immunoreactivity for (**A**) CLDN5 (a marker of tight junctions formed by brain endothelial cells), (**B**) PECAM1 (a marker of endothelial cells), (**C**) GFAP (astrocyte marker), and (**D**) PDGFR-β (pericyte marker). Scale bar: 20 µM.

Additionally, to determine the cellular composition of the isolated BrMV fractions, for which heterogeneity is expected, we prepared immunostaining for CLDN5, PECAM1, astroglial marker GFAP, and brain pericyte marker PDGFRβ (Figure 3A–D, respectively). Strong immunostaining was shown for CLDN5 and PECAM1 in brain capillary endothelial cells (Figure 3A,B), while a rather weak signal was shown for GFAP and PDGFRB (Figure 3C,D).

### 3.2. Selection of Reference Genes and Efficiency of Primers

We selected for our analysis 10 reference genes from the literature, which play various functions in the cell. We took care to include reference genes with expression products present in the cytoplasm, nuclei, or cell membrane. The full names and functions of the selected genes are presented in Table 2, which also demonstrates that mean efficiency values that in almost all cases were higher than 80%, with the exception of *Rplp2*, for which it was 76%. In Table 2, the expression level of analyzed reference genes is represented as the mean of the raw quantification cycle (Cq) ± SEM. The highest expression was demonstrated in all BrMV samples for *Rps18* (Cq ~17.36), which is not surprising as *Rps18* represents the bulk of total RNA, and in all ChP samples for *Ppia* (Cq ~16.37). The Cq values of other candidates were between 18.8 and 26.5 in BrMV and 18.9 and 24 in ChP. The lowest expression was demonstrated for *Hmbs* in BrMV (Cq ~26.5) and *Tbp* in ChP (Cq ~24.08). The largest variation across the studied samples was observed in the expression of *Rplp2* with Cq range of 3.91 in BrMV, and *Apoe* with Cq range of 5.06 in ChP. The smallest variation was observed in the expression of *Rps18* with Cq range of 1.77 in BrMV and *Ywhaz*/*Rps18* with Cq range of 1.86/1.92 in ChP, respectively.

### 3.3. Selection and Verification of the Most Stable Reference Genes for Brain Microvessels

**Figure 4:**
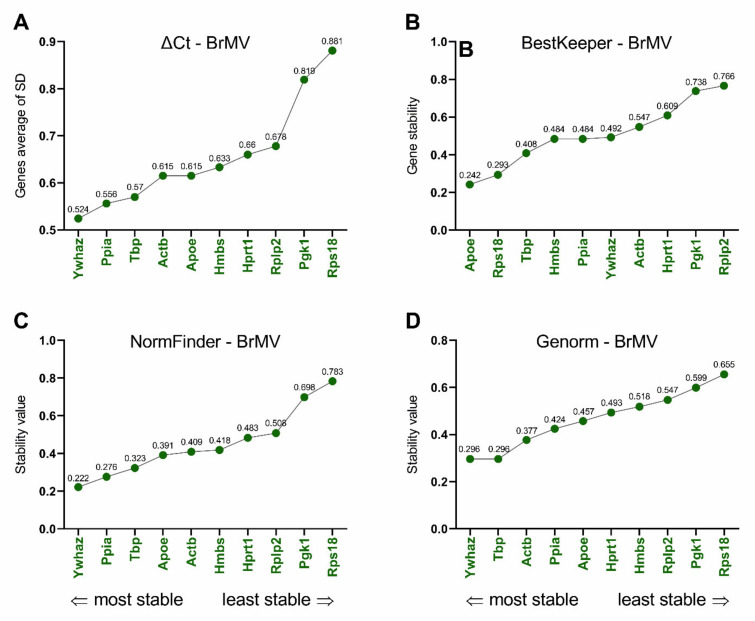
Expression stabilities and ranking of reference genes in rat brain microvessels (BrMV; *n* = 6, 6 females/3 samples and 6 males/3 samples for each time point) according to the ΔCt (**A**), BestKeeper (**B**), NormFinder (**C**), and Genorm (**D**) algorithms. Ranking calculated using RefFinder.

As indicated in Figure 4, the ΔCt method, NormFinder, and Genorm algorithms identified *Ywhaz* as the most stable reference gene for circadian studies in rat BrMV (Figure 4A,C,D). Moreover, the Genorm indicated *Tbp* to be as stable as *Ywhaz* (the stability value for both genes was 0.296). In turn, the BestKeeper method identified *Apoe* as the most stable reference gene (Figure 4B). The ΔCt method, NormFinder, and Genorm identified *Rps18* as the least stable reference gene in circadian studies of rat BrMV, while BestKeeper pointed to *Rplp2* as the least stable. However, all the algorithms used in this work identified *Pgk1* as the second least stable reference gene. The ranges for stability values were as follows: 0.524–0.881 ΔCt, 0.242–0.766 BestKeeper, 0.222–0.783 NormFinder, and 0.296–0.655 Genorm (Figure 4A–D, respectively).

**Figure 5:**
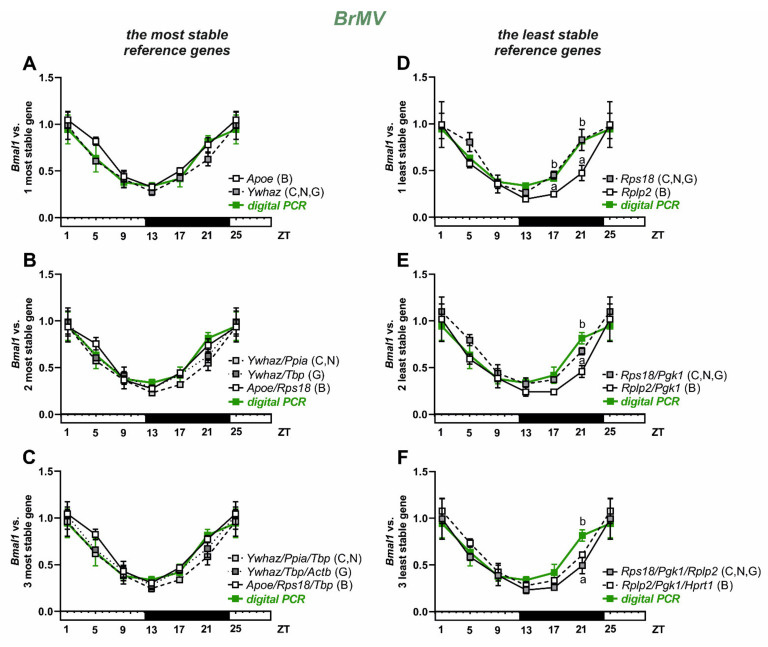
Relative expression of *Bmal1* in isolated rat brain microvessels (BrMV; n = 6, 6 females/3 samples and 6 males/3 samples for each time point). Data from ZT1 and ZT25 (ZT-Zeigeber Time) are double plotted in all panels. Data calculated vs. reference genes selected as the most stable, left panels (**A**–**C**), and the least stable, right panels (**D**–**F**). The additional green plot refers to digital PCR results and different lower case letters indicate significant differences at *p* < 0.05 between qPCR and digital PCR results at a given time point. Abbreviations: C—ΔCt, B—BestKeeper, N—NormFinder, G—Genorm.

The circadian expression of Bmal1 in rat BrMV was analyzed (Figure 5) using reference genes selected as the most or least stable (Figure 4). We used the reference genes individually (*Ywhaz* and *Apoe*; Figure 5A) or in a combination of two (*Ywhaz*/*Ppia*, *Ywhaz*/*Tbp,* and *Apoe*/*Rps18*; Figure 5B) or three (*Ywhaz/Ppia/Tbp*, *Ywhaz/Tbp/Actb,* and *Apoe/Rps18/Tbp*; Figure 5C). The results of *Bmal1* expression analysis, obtained using *Rps18* gene, selected by the ΔCt method, NormFinder, and Genorm as the least stable, both individually (Figure 5D) and in a combination of two least stable genes (*Rps18/Pgk1*, Figure 5E), did not differ significantly from the digital PCR results. In turn, the results obtained using *Rplp2* selected by BestKeeper as the least stable gene differed significantly (*p* > 0.05) from the digital PCR results at ZT17 (Figure 5D) and ZT21 (Figure 5D–F) time points. The analysis with CircWave 1.4 software showed a statistically significant (*p* < 0.05) circadian oscillation of *Bmal1* expression in BrMV analyzed with the use of reference genes selected as both the most stable and the least stable (Supplementary Table S1).

Gender differences in the clock genes’ oscillations in rat ChP were described [10]; therefore, we analyzed the circadian oscillation of *Bmal1* in brain microvessels as follows: (i) all BrMV fractions as well as BrMV isolated separately from (ii) female and (iii) male rats. The results are presented in Supplementary Table S1, which demonstrated that the level of significance of the circadian oscillations was higher for females compared with males, except for the analysis using *Apoe*/*Rps18*/*Tbp*, for which the oscillation was significantly higher for males compared with females, and the analysis using *Rps18*/*Pgk1*, for which the significances were equal for both genders (Supplementary Table S1). In addition, CircWave analysis of the digital PCR results showed a significant circadian oscillation of *Bmal1* expression in BrMV (*p* = 5.4 × 10^−5^, Supplementary Table S3), with this significance being greater in samples from females versus males (*p* = 0.001 vs. *p* = 0.04, respectively, Supplementary Table S3).

### 3.4. Selection and Verification of the Most Stable Reference Genes for Choroid Plexus

**Figure 6:**
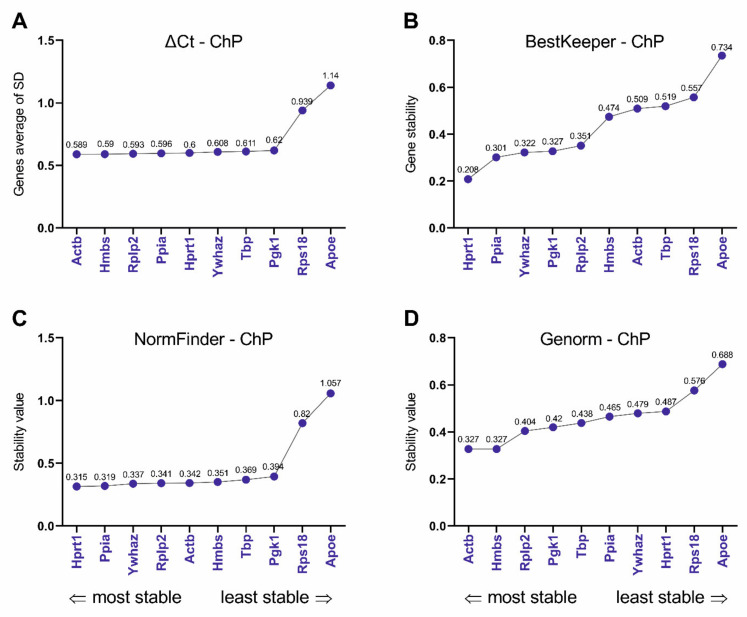
Expression stabilities and ranking of reference genes in the choroid plexus (ChP; *n* = 6, 6 females/3 samples and 6 males/3 samples for each time point) according to the ΔCt (**A**), BestKeeper (**B**), NormFinder (**C**), and Genorm (**D**) algorithms. Ranking calculated using RefFinder.

As indicated in Figure 6, the ΔCt method and Genorm algorithm identified *Actb, Hmbs,* and *Rplp2*, in the presented order, as the most stable reference genes for circadian studies in rat ChP (Figure 6A,D). Moreover, Genorm indicated *Actb* and *Hmbs* as identically stable (the stability value for both genes was 0.327). In turn, BestKeeper and NormFinder identified *Hprt1/Ppia/Ywhaz*, in the presented order, as the most stable reference genes (Figure 6B,C). All used algorithms identified *Apoe* and *Rps18*, in the presented order, as the least stable reference genes in circadian studies in rat ChP. The ranges for stability values were as follows: 0.589–1.14 ΔCt, 0.208–0.734 BestKeeper, 0.315–1.057 NormFinder, and 0.327–0.688 Genorm (Figure 6 A–D, respectively).

**Figure 7:**
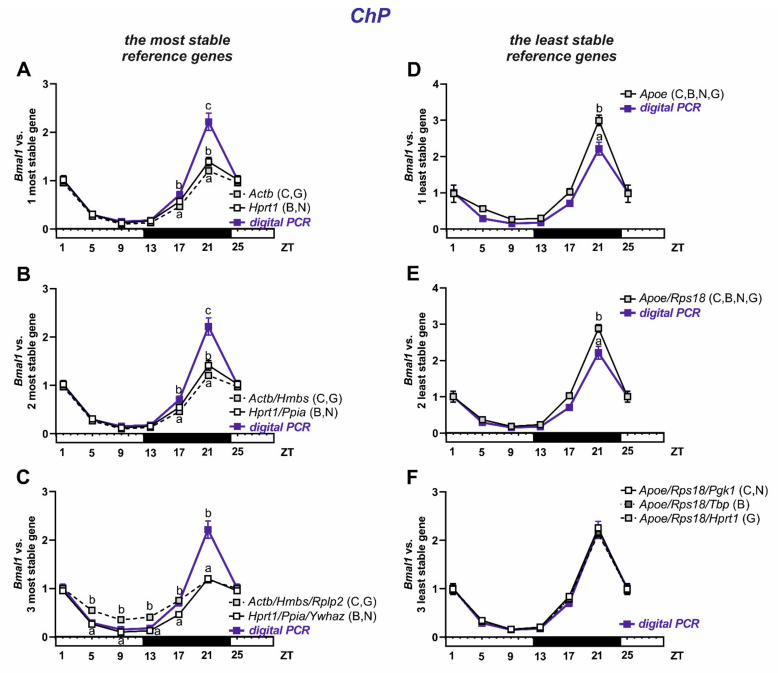
Relative expression of *Bmal1* in isolated rat choroid plexus (ChP; *n* = 6, 6 females/3 samples and 6 males/3 samples for each time point). Data from ZT1 and ZT25 (ZT-Zeigeber Time) are double plotted in all panels. Data calculated vs. reference genes selected as the most stable, left panels (**A**–**C**), and the least stable, right panels (**D**–**F**). The additional blue plot refers to digital PCR results and different lower case letters indicate significant differences at *p* < 0.05 between qPCR and digital PCR results at a given time point. Abbreviations: C—ΔCt, B—BestKeeper, N—NormFinder, G—Genorm.

In the circadian expression of *Bmal1* gene in rat ChP (Figure 7), we found in almost all analyses significant differences (*p* > 0.05) as compared with digital PCR results at least for one time-point—ZT21. *Bmal1* expression was lower for the most stable genes (Figure 7A–C) and higher for one or two of the least stable genes (Figure 7D,E). The only exception was the analysis using combinations of three reference genes selected as the least stable ones (*Apoe/Rps18/Pgk1*, *Apoe/Rps18/Tbp*, and *Apoe/Rps18/Hprt1*; Figure 7F), where no differences were found. In addition, a significantly lower expression compared with the digital PCR results was observed at the ZT17 point, using the *Actb*, a combination of two: *Actb/Hmbs* and three: *Hprt1/Ppia/Ywhaz* genes, all listed as the most stable (Figure 7A–C). Using the *Actb/Hmbs/Rplp2* combination resulted in a significantly higher expression (*p* < 0.05) of *Bmal1* compared with the digital PCR results at points ZT5, ZT9, and ZT13. The analysis with CircWave showed a statistically significant (*p* < 0.05) circadian oscillation of *Bmal1* expression in rat ChP analyzed using reference genes selected as both the most stable and the least stable (Supplementary Table S2).

As gender variations in circadian oscillation in ChP were described previously [10], we analyzed the expression of *Bmal1* in rat ChP collected from (i) all animals as well as separately from (ii) female or (iii) male rats (Supplementary Table S2). Using reference gene combinations, the significance levels were as follows: we found higher levels in female compared with male samples for *Hprt1*, *Apoe, Hprt1/Ppia*, and *Apoe/Rps18/Tbp*; a similar level for *Actb/Hmbs*, *Hprt1/Ppia/Ywhaz*, *Apoe/Rps18/Pgk1*, and *Apoe/Rps18/Hprt1;* and higher levels in male compared with female samples for *Actb* and *Actb/Hmbs/Rplp2* (Supplementary Table S2). In addition, CircWave analysis of the digital PCR results showed a significant circadian oscillation of *Bmal1* expression in ChP (*p* = 0, Supplementary Table S3), with a greater statistical significance in females versus males (*p* = 4.7 × 10^−5^ vs. *p* = 0.001, respectively, Supplementary Table S3).

## 4. Discussion

The method of the first choice for quantitative gene expression analysis is qPCR, which is fast, accurate, economical, and can be easily implemented in a laboratory [15]. However, it is important to normalize qPCR data to compensate for the variability that can appear at all stages, from the quantity and quality of RNA, through reverse transcription to the efficiency of the PCR reaction, and using reference genes [33]. Owing to the high sensitivity of qPCR, normalization with stable reference genes is important for accurate analysis of the biological variation in the data. The application of non-validated reference genes can lead to inaccurate data interpretation and misleading conclusions [34]. Unfortunately, there is no ideal reference gene, or even a combination of genes, that applies to all types of cells, tissues, species, and experimental conditions. Clearly, it is necessary to identify and validate the method most appropriate for particular experimental conditions. Importantly, in circadian studies, the expression of selected reference genes must not oscillate over the clock. Therefore, we present for the first time the selection of reference genes for the circadian analysis of mRNA expression in rat BrMV and ChP, along with verification of the selection.

In two of our previous studies, using a sheep model with melatonin implants (mimicking short days) and control animals (long days), we considered the effect of the photoperiod on ChP functioning. In this study, we used combinations of three reference genes: glyceralaldehyde-3-phosphate dehydrogenase (*Gapdh*), histone deacetylase 1 (*Hdac1*), and succinate dehydrogenase A (*Sdha*) [35], as well as a combination where *Sdha* was replaced by *Actb* [36]. This selection was based on our previous experience using the same sheep model, where we used *Gapdh*, *Hdac1*, and *Actb* [37,38]. In the current research, we did not take into account the genes *Hdac1* and *Sdha*, which are often used in studies on tissues from large farm animals, like bovine kidney and sheep neutrophils, where *Gapdh* and *Sdha* were selected as most stable [39,40], as well as the bovine oviductal epithelial cells, for which *Hdac1* was selected [41]. In this work we have selected 10 reference genes previously reported in different studies on rodent tissues; e.g., [30,42]. Moreover, we took into account genes whose expression products have different functions and are located in different cell compartments (Table 2). We did not analyze *Gapdh*, often used as a reference gene. In preliminary experiments, we tested several primer pairs designed for rat *Gapdh*, but we observed additional products in regular PCR. Our results may be connected to the fact that the genome of rats contains 329 *Gapdh* pseudogenes, similarly to humans (60 pseudogenes) and mice (285 pseudogenes) [43]. Some of the *Gapdh* pseudogenes are expressed and have nearly identical sequences to the target transcript; therefore, primers may detect the presence of the pseudogenes along with the cDNA of the active transcript. It should be mentioned that we did not encounter similar problems when analyzing sheep tissue. Moreover, we realize that the selection of 10 reference genes is a limitation of our study and we cannot exclude that other reference genes could be equally or more stable in BrMV and ChP samples collected around the clock. It should be noted that the order of Cq differs between the BrMV and ChP samples. For example, the reference genes expressed at the highest levels in the BrMV samples were *Rps18* (17.36), *Actb* (19.56), and *Apoe* (19.58), while in ChP, they were *Ppia* (16.37), *Actb* (18.93), and *Rps18* (19.22). The reference genes expressed at the lowest levels were *Hmbs* (26.5), *Tbp* (25.74), and *Hprt1* (23.46) in the BrMV samples and *Tbp* (24.08), *Hmbs* (23.92), and *Apoe* (22.77) in the ChP samples. Considering the fact that the expression of reference genes can vary between tissues [44], the optimal solution is to use those showing minimal variability between tissues [45]. Therefore, *Ywhaz* with Cq of 20.74 and 20.41 in the BrMV and ChP, respectively, could be used to compare the expression between these tissues in rats.

Our research was carried out in three stages: verification of the isolation of BrMV and ChP, selection of the most stable reference genes for circadian studies on these brain barrier samples, and verification of this selection by analyzing the *Bmal1* expression using the qPCR and digital PCR methods. The selection of *Bmal1* was based on previous reports of its expression and circadian oscillation in the ChP and brain endothelium [10,12,13]. Just recently, Zhang et al. [46] confirmed the important role of *Bmal1* in the regulation of intracellular magnesium levels and efflux transport of xenobiotics in human brain endothelial cells. In the first stage, we confirmed the predominant presence of endothelial cells in BrMV fractions, characterized by the very high expression of Pecam1 marker [47] at mRNA and protein levels. We also confirmed the presence of the BBB tight junctions in BrMV, as indicated by the high expression of its key component, Cldn-5 [48], at the mRNA and protein levels (strong immunostaining). These results are in line with a previous work describing *Pecam1* and *Cldn-5* as the most relevant BrMV marker genes [21]. *Plvap* is an indicator for fenestrated blood vessels [21], and we used it as a marker gene for ChP. *Plvap* mRNA expression in our samples confirmed that BrMV samples were not contaminated by fenestrated vessels and verified the quality of ChP samples. The protocol of ChP isolation was additionally verified by the high mRNA expression of the transthyretin gene (Cq = 9.3, data not shown), considered as a marker gene for ChP epithelial cells [49]. Brain capillaries are mainly composed of cerebral endothelial cells, but also contain pericytes embedded in the basal membrane and are surrounded by astroglial endfeet [50]. In our BrMV samples, weak immunostaining was observed for the astrocytic marker GFAP [51] and the pericyte marker PDGFRβ [52] as compared with the endothelial marker proteins, indicating that the samples contained mostly brain endothelial cells.

Depending on the algorithm used, various reference genes were selected as the most stable. For BrMV, the softwares ΔCT, NormFinder, and Genorm selected *Ywhaz* alone or together with the next two most stable, that is, *Ppia/Tbp* according to ΔCt and NormFinder or *Tbp/Actb* according to Genorm. BestKeeper selected *Apoe* as the most stable, followed by *Rps18* and *Tbp*. It should be noted that *Rps18* was consistently indicated by other algorithms as the least stable, despite that this gene had the highest expression level in BrMV (Cq = 17.36) and the lowest differences within the samples (Cq range of 1.77). Importantly, our verification of the suitability of reference genes by analyzing the expression of the *Bmal1* shows that the use of both genes selected as the most stable as well as *Rps18* or the *Rps18*/*Pgk1* combination considered the least stable led to results that did not differ from the digital PCR data. This indicates these genes are sufficiently stable for circadian studies. It should be noted that digital PCR is a method that does not use reference genes, hence it is a good choice for validation for performing several qPCR analyses on a given experimental model [53]. For ChP, ΔCT and Genorm selected *Actb*/*Hmbs*/*Rplp2* as the most stable reference genes, while NormFinder and BestKeeper selected *Hprt1/Ppia/Ywhaz*. However, for this tissue, the results of *Bmal1* expression analysis using the most and least stable reference genes differed from the digital PCR results, at least at one time-point, ZT21, where the highest expression was seen. Interestingly, this observation does not apply to the analysis using the three genes identified as least stable, which include *Apoe*/*Rps18*, according to all algorithms, and *Pgk1*, *Tbp,* or *Hprt1*, according to ΔCt and NormFinder, BestKeeper, and Genorm, respectively. In this case, irrespective of which third least stable gene was used, the results were in line with those from digital PCR. The concordance of the qPCR and digital PCR results was noticed after adding *Rps18* to the analysis, which was characterized by stable expression and its Cq ratio was 1.92. Therefore, as we observed for BrMV, *Rps18* seems to be the right choice as a reference gene for ChP for circadian studies. In accordance with our data, significant effects of the time of day on most housekeeping reference genes were demonstrated in the liver, except for *Rps18* [54]. For the selection of reference genes in addition to circadian rhythm, basic parameters such as the level of expression (Cq) should be taken into account. Cq values should not strongly differ from the genes under study. Moreover, a parameter such as Cq range, which for *Rps18* was 1.77 in BrMV and 1.92 in ChP, should be also considered. Additionally, we observed that *Apoe*, selected as one of the most stable genes for BrMV, was unanimously selected as the least stable for ChP, where its Cq range was 5.06, which indicates the need to select reference genes taking into account not only the species and the experimental conditions, but also the kind of tested tissue. Our data on significant circadian fluctuations of *Bmal1* expression in BrMV and ChP, observed in all analyses, indicate that a wide range of reference genes can give reliable results. We can conclude that, in circadian studies regarding BrMV and ChP, almost all of the presented candidate genes (Table 2) may provide correct results. This suggestion is in line with the work of Hellemans et al. [55], where a Genorm stability value up to 1 indicates stable reference gene for more heterogeneous panels. According to Genorm guidelines, stability values should not exceed 0.75, while in our studies, all the stability values determined by Genorm meet this requirement, with a maximum of 0.655 in the BrMV and 0.688 in the ChP (Figure 4D and Figure 6D, respectively). We would like to note that, when relatively subtle circadian fluctuations are expected, it is worth considering the use of the digital PCR method. However, it is still a relatively expensive and time-consuming method, which is used mostly when precise results are needed, like in clinical diagnostics [56,57,58].

The original goal of our study was not to analyze sex differences, but we included both male and female rats in our research. Therefore, we additionally analyzed the circadian oscillation for each sex separately and noticed, in many cases of qPCR analyses as well as in digital PCR, the higher statistical significance of *Bmal1* oscillations in females compared with males. Depending on the reference genes’ combination, we also observed higher oscillation significance for males than females: *Apoe*/*Rps18*/*Tbp* in BrMV; *Actb* and *Actb*/*Hmbs*/*Rplp2* in ChP. The sex difference between expression level of these genes may be explained by the facts that estrogen regulates the expression of some reference genes, for example, *Actb* [59], and that the plasma concentration of estradiol is much lower in male than female rodents [60]. Nevertheless, our observation regarding *Bmal1* expression is similar to that made by Quintela et al. [10], except that in this study, no significant oscillation was found in ChP from males. This discrepancy does not change the fact that our research confirms the observation that the circadian expression of clock genes may differ between males and females. For that reason, we strongly suggest conducting circadian studies on both sexes separately, at least regarding brain barriers.

## 5. Conclusions

We identified for circadian studies as the most stable reference genes *Ywhaz* and *Apoe* for brain microvessels and *Actb* and *Hprt1* for choroid plexus based on RefFinder selection. The results of this selection were verified by analysis of the circadian gene *Bmal1* expression. We found that *Rps18* is also a suitable reference gene and observed that the use of at least two reference genes provided more reliable results. Moreover, most of the ten tested reference genes could be successfully used owing to the demonstrated significant circadian oscillations of the *Bmal1* expression using both the most and the least stable genes. Additionally, we demonstrated sex differences in the statistical significance of *Bmal1* oscillations, thus we recommend separate studies for both sexes at least for over-the-clock changes in studies on brain barriers.

## Figures and Tables

**Table 2:** Reference genes analyzed in brain cortical microvessel and choroid plexus samples: gene names, functions, the efficiency of qPCR reaction, and raw quantification cycle values.

Gene Symbol	Full Name	Function	Primers Efficiency	BrMV Cq Mean ± SEM	BrMV Cq Range	ChP Cq Mean ± SEM	ChP Cq Range
***Actb***	Beta actin	cytoskeletal structure protein	85%	19.56 ± 0.13	3.25	18.93 ± 0.08	2.12
***Apoe***	Apolipoprotein E	transport of lipids, fat soluble vitamins, and cholesterol	85%	19.58 ± 0.08	1.95	22.77 ± 0.2	5.06
***Hmbs***	Hydroxymethylbilane synthase	the third enzyme in the heme biosynthetic pathway	85%	26.5 ± 0.13	3.16	23.92 ± 0.09	2.36
***Hprt1***	Hypoxanthine-guanine phosphoribosyltransferase	recycle purines in cells	86%	23.43 ± 0.14	3.74	20.66 ± 0.07	2.20
***Pgk1***	Phosphoglycerate kinase 1	enzyme involved in glycolysis process	86%	22.42 ± 0.13	3.02	19.63 ± 0.1	3.08
***Ppia***	Peptidylprolyl isomerase A	catalyse the cis–trans isomerisation of peptide bonds N-terminal to proline residues in polypeptide chains	84%	18.82 ± 0.12	3.33	16.37 ± 0.07	2.15
***Rplp2***	Ribosomal protein lateral stalk subunit P2	ribosomal phosphoprotein—component of the 60S subunit	76%	22.27 ± 0.14	3.91	21.71 ± 0.08	2.27
***Rps18***	Ribosomal protein S18	ribosomal subunit	83%	17.36 ± 0.07	1.77	19.22 ± 0.08	1.92
***Tbp***	TATA-box binding protein	transcription factor	87%	25.74 ± 0.15	2.78	24.08 ± 0.08	2.37
***Ywhaz***	Tyrosine 3-monooxygenase/tryptophan 5-monooxygenase activation protein zeta	involved in signal transduction pathways and role in tumor progression	90%	20.74 ± 0.11	2.56	20.41 ± 0.08	1.86

Efficiency ChP/BrMV—mean efficiency of qPCR reaction calculate using the “Real-Time PCR Miner” (online software) for qPCR at ChP (choroid plexus) and BrMV (brain microvessels) cDNA; Cq—quantification cycle values from real-time PCR.

## Data Availability

Data are available from the corresponding author upon reasonable request.

## Supplementary Materials

The following are available online at https://www.mdpi.com/article/10.3390/biom11081227/s1, Table S1: Circadian oscillation of Bmal1 expression in brain microvessels analyzed by CircWave; Table S2: Circadian oscillation of Bmal1 expression in choroid plexus analyzed by CircWave; Table S3: Circadian oscillation of Bmal1 expression analyzed using digital PCR.
