# Supplementary material for: Identification of Reference Genes for Circadian Studies on Brain Microvessels and Choroid Plexus Samples Isolated from Rats
Source: Biomolecules. 2021 Aug 17;11(8):1227. doi: 10.3390/biom11081227 (PMC8394446; doi:10.3390/biom11081227)
Supplement: Supplementary file 1 [file biomolecules-11-01227-s001.zip › biomolecules-1306152-supplementary.pdf]

Identification of reference genes for circadian studies on brain microvessels and choroid plexus samples isolated from rats

Aleksandra Szczepkowska, András Harazin, Lilla Barna, Mária A. Deli and Janina Skipor

Supplementary Materials

Supplementary Table S1 Circadian oscillation of *Bmal1* expression in brain microvessels analysed by CircWave

BrMV - *Bmal1* vs. reference genes combinations			r^2	*p*-value
most stable genes	vs. *Ywhaz*	all	0.569	1.2E-05
		females	0.784	1E-05
		males	0.602	0.01
	vs. *Apoe*	all	0.845	0
		females	0.883	1E-07
		males	0.812	0.0005
	vs. *Ywhaz/Ppia*	all	0.558	1.6E-05
		females	0.699	0.0001
		males	0.592	0.02
	vs. *Ywhaz/Tbp*	all	0.586	6.7E-06
		females	0.805	4.7E-06
		males	0.602	0.016
	vs. *Apoe/Rps18*	all	0.732	0
		females	0.845	1E-06
		males	0.645	0.009
	vs. *Ywhaz/Ppia/Tbp*	all	0.593	5E-06
		females	0.769	1.7E-05
		males	0.616	0.01
	vs. *Ywhaz/Ppia/Actb*	all	0.615	2.5E-06
		females	0.835	1.3E-06
		males	0.604	0.01
	vs. *Apoe/Rps18/Tbp*	all	0.891	0
		females	0.967	0.006
		males	0.842	0.0002
least stable genes	vs. *Rps18*	all	0.727	0
		females	0.804	5E-06
		males	0.792	0.0008
	vs. *Rplp2*	all	0.488	0.0002
		females	0.677	0.0002
		males	0.505	0.04
	vs. *Rps18/Pgk1*	all	0.807	0.046
		females	0.957	0.01
		males	0.611	0.01
	vs. *Rplp2/Pgk1*	all	0.469	0.0002
		females	0.746	3.5E-05
		males	0.495	0.05

	vs. *Rps18/Pgk1/Rplp2*	all	0.705	1E-07
		females	0.914	0
		males	0.612	0.01
	vs. *Rplp2/Pgk1/Hprt1*	all	0.486	0.0001
		females	0.689	0.0001
		males	0.522	0.04

BrMV – brain microvessels; "0"- very low p-value; all genes abbreviations are explained in the main manuscript

Supplementary Table S2 Circadian oscillation of *Bmal1* expression in choroid plexus analysed by CircWave

**ChP - *Bmal1* vs. reference genes combinations**			r^2	***p*-value**
**most stable genes**	vs. *Actb*	all	0.975	0.03
		females	0.422	0.02
		males	0.986	1.4E-06
	vs. *Hprt1*	all	0.935	0
		females	0.932	2E-07
		males	0.945	0.02
	vs. *Actb/Hmbs*	all	0.977	0
		females	0.985	1.1E-05
		males	0.976	9.3E-06
	vs. *Hprt1/Ppia*	all	0.932	7E-06
		females	0.927	3E-07
		males	0.958	6.5E-05
	vs. *Actb/Hmbs/Rplp2*	all	0.970	0.0003
		females	0.971	0.03
		males	0.989	5E-07
	vs. *Hprt1/Ppia/Ywhaz*	all	0.985	0
		females	0.986	3E-06
		males	0.989	1.4E-06
**least stable genes**	vs. *Apoe*	all	0.868	1.4E-05
		females	0.854	2.4E-05
		males	0.899	0.001
	vs. *Apoe/Rps18*	all	0.927	0
		females	0.933	2E-07
		males	0.918	0.0006
	vs. *Apoe/Rps18/Pgk1*	all	0.952	0
		females	0.959	0.0001
		males	0.947	0.0001
	vs. Apoe/Rps18/Tbp	all	0.950	0
		females	0.959	8.3E-05
		males	0.944	0.0002
	vs. *Apoe/Rps18/Hprt1*	all	0.950	0
		females	0.952	0.0001
		males	0.953	9.6E-05

ChP – choroid plexus; "0"- very low p-value; all genes abbreviations are explained in the main manuscript

Supplementary Table S3 Circadian oscillation of Bmal1 expression analysed using digital PCR.

***Bmal1* expression analysed using digital PCR**		**r^2**	***p*-value**
**BrMV**	all	0.607	5.4E-05
	females	0.780	0.001
	males	0.493	0.04
**ChP**	all	0.922	0
	females	0.962	4.7E-05
	males	0.905	0.001

BrMV – brain microvessels; ChP – choroid plexus; "0"- very low p-value
